# Wear Characteristics of Metallic Biomaterials: A Review

**DOI:** 10.3390/ma8052749

**Published:** 2015-05-21

**Authors:** Mohamed A. Hussein, Abdul Samad Mohammed, Naser Al-Aqeeli

**Affiliations:** 1 Department of Mechanical Engineering, King Fahd University of Petroleum & Minerals, Dhahran 31261, Saudi Arabia; E-Mails: mahussein@kfupm.edu.sa (M.A.H.); naqeeli@kfupm.edu.sa (N.A.-A.); 2 Department of Mechanical Engineering, Kafrelsheikh University, Kafrelsheikh 33516, Egypt

**Keywords:** metallic biomaterial, tribology, wear, friction

## Abstract

Metals are extensively used in a variety of applications in the medical field for internal support and biological tissue replacements, such as joint replacements, dental roots, orthopedic fixation, and stents. The metals and alloys that are primarily used in biomedical applications are stainless steels, Co alloys, and Ti alloys. The service period of a metallic biomaterial is determined by its abrasion and wear resistance. A reduction in the wear resistance of the implant results in the release of incompatible metal ions into the body that loosen the implant. In addition, several reactions may occur because of the deposition of wear debris in tissue. Therefore, developing biomaterials with high wear resistance is critical to ensuring a long life for the biomaterial. The aim of this work is to review the current state of knowledge of the wear of metallic biomaterials and how wear is affected by the material properties and conditions in terms of the type of alloys developed and fabrication processes. We also present a brief evaluation of various experimental test techniques and wear characterization techniques that are used to determine the tribological performance of metallic biomaterials.

## 1. Introduction

For decades, metals have been used extensively in a variety of applications in the medical field. Specifically, metals are used for internal support and biological tissue replacements, such as joint replacements, dental roots, orthopedic fixation, and stents [1]. The metals and alloys that are primarily used in biomedical applications are stainless steels, Co alloys, and Ti alloys [2,3].

All biomaterials are required to satisfy various criteria, such as adequate strength, high resistance to corrosion, bioadhesion, biofunctionality, biocompatibility, high wear resistance, and low friction [4]. However, the various biomaterials that have been developed thus far do not satisfy all of the above requirements. Wear and corrosion have been reported to be the primary reasons for the failure of implant elements. Some of the applications of tribology in the biomedical field are as follows: wear of dentures [5,6], heart valves [7], plates and screws in bone fracture repair [8]; friction between garments and friction between garments and skin [9,10]; and lubrication of artificial heart pumps [11], pleural surfaces, and the pericardium. Wear is a major factor in controlling and determining the long-term clinical performance of a metallic biomaterial.

Hence, the objective of this work is to address the current state of knowledge of the wear of metallic biomaterials and how wear is affected by material properties and conditions in terms of the type of alloys developed, fabrication processes, experimental test techniques, and characterization methodologies.

## 2. Desired Properties of Biomaterials

A biomaterial should satisfy the criteria given below.

- **Mechanical properties:** Stress shielding can be prevented by matching the modulus of elasticity of biomaterials to that of bone, which varies from 4 to 30 GPa [12,13]. Additionally, the material should have a low modulus combined with high strength to prolong the service period of the implant and prevent loosening, thereby preventing the need for revision surgery.
- **Biocompatibility:** The developed material should be compatible with living systems and not cause any bodily harm, which includes all of the negative effects a material can have on the components of a biological system (bone, extra- and intracellular tissues, and ionic composition of plasma) [11,12,13].
- **High wear resistance:** The material should have a high wear resistance and exhibit a low friction coefficient when sliding against body tissues. An increase in the friction coefficient or a decrease in the wear resistance can cause the implant to loosen [14,15]. Moreover, the wear debris generated can cause inflammation that is destructive to the bone supporting the implant.
- **High corrosion resistance:** An implant that is made of a biomaterial with a low corrosion resistance can release metal ions into the body, which in turn produces toxic reactions [16].
- **Osseointegration:** Osseointegration was first defined as “a direct structural and functional connection between ordered, living bone and the surface of a load-carrying implant” [17]. The roughness, chemistry, and topography of the surface play a major role in good osseointegration [18]. Implant loosening results from the non-integration of the implant surface into the adjacent bone [19]. Few researchers mention that osseiontegration is undesirable due to the risk of not being able to remove the implant after use [20]. However, a few of them have also demonstrated that the implant could be removed safely [20]. Thus osseointegration is a desirable property for a biomaterial in some applications such as in implant where it is to be made sure that the implant will integrate properly with the bone and other tissues [21].
- **Non-toxic:** The material should be neither genotoxic (which can alter the DNA of the genome) nor cytotoxic (causes damage to individual cells).
- **Long fatigue life:** The material should exhibit a high resistance to failure by fatigue to prevent implant failure and stress shielding from fatigue fracture. The failure of implants by fatigue has been reported for hip prostheses [22].

## 3. Types of Biomaterials

The materials that are used to build biomedical devices (orthopedic, dental, bone cements, *etc*.) can be classified into metallic materials, ceramics, polymers, and composites. Metallic materials within these four categories, despite some shortcomings, such as the release of metallic ions and wear debris, are widely used due to their high strength, toughness, and good biocompatibility.

### 3.1. Metallic Alloys for Biomaterials

The high reliability of metallic biomaterials, in terms of their mechanical performance, has resulted in their use “mainly for the fabrication of medical devices for the replacement of hard tissue such as artificial hip joints, bone plates, and dental implants” [2]. Multiple types of materials and alloys have been investigated in the medical field for their various properties and characteristics [1]. Different alloy systems have been developed for use in the medical field, including stainless steels, Co alloys, and Ti alloys. Table 1, Table 2 and Table 3 summarize the chemical composition of alloys that are registered in the ASTM Standard and have been developed for biomedical applications [23]. A brief description of each material is given below.

**Table 1:** Titanium alloys for biomedical applications [23].

Alloy	Microstructure
1. Pure Ti	(ASTM F67-89)
2. Ti-6Al-4V ELI (ASTM F136-84, F620-87)	α+β type
3. Ti-6Al-4V (ASTM F1108-88)	α+β type
4. Ti-6Al-7Nb (ASTM F1295-92, ISO5832-11)	α+β type(Swiss)
5. Ti-5Al-2.5Fe (ISO5832-10)	α+β type (Germany)
6. Ti-5Al-3Mo-4Zr	α+β type (Japan)
7. Ti-15Sn-4Nb-2Ta-0.2Pd	α+β type (Japan)
8. Ti-15Zr-4Nb-2Ta-0.2Pd	α+β type (Japan)
9. Ti-13Nb-13Zr (ASTM F1713-96)	near β type (U.S.A.), Low modulus
10. Ti-12Mo-6Zr-2Fe (ASTM F1813-97)	near β type (U.S.A.), Low modulus
11. Ti-15Mo	β type (U.S.A.), Low modulus
12. Ti-16Nb-10Hf	β type (U.S.A.), Low modulus
13. Ti-15Mo-5Zr-3Al	β type (Japan), Low modulus
14. Ti-15Mo-2.8Nb-0.2Si-0.26O	β type (U.S.A.), Low modulus
15. Ti-35Nb-7Zr-5Ta	β type (U.S.A.), Low modulus
16. Ti-29Nb-13Ta-4.6Zr	β type (Japan), Low modulus
17. Ti-40Ta, Ti-50Ta	β type (U.S.A), High corrosion resistance

**Table 2:** Chemical compositions of stainless steels registered in ASTM standard for biomedical applications [23].

ASTM designation	Alloy	Cr	Ni	Mo	N	Mn	C	P	S	Si	Cu	Fe
(F138-92)	Bar and Wire											
	Grade 1	17.00–19.00	13.00–15.50	2.00–3.00	−0.1	−2.0	−0.08	−0.025	−0.01	−0.75	−0.5	balance
	Grade 2	17.00–19.00	13.00–15.50	2.00–3.00	−0.1	−2.0	−0.03	−0.025	−0.01	−0.75	−0.5	balance
(F139-96)	18Cr-14Ni-2.5Mo	17.00–19.00	13.00–15.00	2.25–3.00	−0.1	−2.0	−0.03	−0.025	−0.01	−0.75	−0.5	balance
(F621-92)	Sheet and Strip	Same chemical composition as specified in Specification F138, grade 1 and 2										
(F1314-95)	Forgings Nitrogen strengthened	20.5–23.5	11.5–13.5	2.0–3.0	0.2–0.4	4.0–6.0	−0.03	−0.025	−0.01	−0.75	−0.5	balance
	22Cr-12.5Ni-5Mn-2.5Mo	(0.10 < Nb < 0.30, 0.10 < V < 0.30)										
(F1586-95)	Bar and Wire											
	Nitrogen strengthened											
	21Cr-10Ni-3Mn-2.5Mo											
		0.25 < Nb < 0.80										

**Table 3:** Chemical compositions of Co alloys registered in ASTM standard for biomedical applications [23].

ASTM designation	Alloy	Cr	Mo	Ni	W	Fe	Ti	C	Si	P	S	Mn	Co
(F75-92)	Co-Cr-Mo Cast alloy	27.0–30.0	5.0–7.0	−1.0		−0.75		−0.35	−1.0			−1.0	balance
(F90-96)	Co-20Cr-15W-10Ni Wrought alloy	19.0–21.0		9.0–11.0	14.0–16.0	−3.0		0.05–0.15	−0.4	−0.03	−0.03	1–2	balance
(F562-95)	Co-35Ni-20Cr-10Mo	19.0–21.0	9–10.5	33.0–37		−1.0	−1.0	0.025	−0.15	−0.015	−0.01	−0.15	balance
	Wrought alloy	(B < 0.0015)											
(F563-95)	Co-Ni-Cr-Mo-W-Fe Wrought alloy	18–22	3–4	15–25	3–4	4–6	0.5–3.5	0.05	0.5		0.01	1.0	balance
(F799-96)	Co-28Cr-6Mo forgings	26.0–30.0	5–7	−1.0		−0.75		−0.35	-1.0			−1.0	balance
(F1058-91)	Co-Cr-Ni-Mo-Fe Wrought alloy												
	Grade 1	19.0–21.0	6.0–8.0	14.0–16.0		balance		0.15	−1.2	−0.015	−0.015	1.5–2.5	39.0–41
		(Be < 0.01)											
	Grade 2	18.5–21.5	6.5–7.5	15.0–18.0		balance		0.15	−1.2	−0.015	−0.015	1.0–2.0	39.0–42
		(Be < 0.001)											
(F1537-94)	Co-28Cr-6Mo Wrought alloy	26.0–30.0	5.0–7.0	−1.0		−0.75		0.35	−1.0			1.0	balance
		(N < 0.25)											

#### 3.1.1. Ti Alloys

The high biocompatibility of Ti and Ti alloys has resulted in their preferential use over other alloy systems in the medical and dentistry fields [24,25,26]. The primary characteristics of Ti alloys that have resulted in their being one of the main choices in the biomedical field include good mechanical properties, excellent corrosion behavior because of a TiO_2_ solid oxide layer, good biocompatibility, a relatively low Young’s modulus, light weight, and non-magnetic behavior. The aforementioned characteristics make Ti and Ti alloys the preferred choices for implantation. However, Ti alloys exhibit poor tribological properties [27] because of “low resistance to plastic shearing, low work hardening, and low protection exerted by surface oxides” [27].

#### 3.1.2. Stainless Steels

The austenitic stainless steel SUS 316L is the only reported stainless steel that is used in the biomedical field. However, a few researchers have found that the Ni contained in this alloy causes allergic reactions [2]. Moreover, pitting, crevice, and stress corrosion have been reported for implants fabricated from SUS 316L [23]. To prevent Ni allergic reactions, an austenitic stainless steel with high nitrogen content has been developed. Therefore, the new research trend is to develop Ni-free stainless steels.

#### 3.1.3. Co Alloys

The wear resistance of Co alloys is higher than that of both Ti alloys and stainless steel alloys [23]. In artificial hip joints, the head of the joint is subjected to wear. Thus, hip joints have been fabricated from Co alloys, such as Co-Cr-Mo alloys, which exhibit high strength and ductility. Dispersing carbide in Co alloys has been reported to increase the resistance to wear of these alloys [2]. Furthermore, the transformation of the metastable γ phase to the ε martensitic phase (via a deformation-induced transformation) has been found to improve the wear resistance of Co alloys [2]. Compared to cast Co-Cr alloys, wrought Co-Cr alloys can be used for implant devices with high strength requirements. However, the Ni content in wrought Co-Cr alloys causes allergic reactions [23].

Some of the mechanical properties of metallic biomaterials are compared in Table 4. Examples of the metallic alloys used in biomedical applications, their advantages and disadvantages are summarized in Table 5.

As observed in Table 4, the Young’s Modulus of Co-Cr alloys and stainless steel is found to be 10 × that of the bone, which may cause stress shielding. However, the Young’s modulus of titanium and its alloys is approximately 0.5× that of stainless steel, and hence the risk of stress shielding is less in titanium and its alloys compared to that of Co-Cr alloys and stainless steel.

**Table 4:** Comparison of mechanical properties of metallic biomaterials with bone [28].

Material	Young’s Modulus, E (GPa)	Yield Strength, (MPa)	Tensile Strength (MPa)	Fatigue Limit, (MPa)
**Stainless steel**	190	221–1213	586–1351	241–820
**Co-Cr alloys**	210–253	448–1606	655–1896	207–950
**Titanium (Ti)**	110	485	760	300
**Ti-6Al-4V**	116	896–1034	965–1103	620
**Cortical bone**	15–30	30–70	70–150	

**Table 5:** Comparison of metallic biomaterials used in the human body.

Metals and alloys	Selected examples	Advantages	Disadvantages	Principal applications [29]
Titanium-based Alloys	CP-Ti, Ti-Al-V, Ti-Al-Nb, Ti- 13Nb-13Zr, Ti-Mo-Zr-Fe	High biocompatibility [24,25,26]. Low Young’s modulus excellent corrosion resistance, low density	Poor tribological properties [27], Toxic effect of Al and V on long term	Bone and joint replacement, fracture fixation, dental implants, pacemaker encapsulation
Cobalt and Cr alloys	Co-Cr-Mo, Cr-Ni-Cr-Mo	High wear resistance [20]	Allergy consideration with Ni, Cr and Co [2] much higher modulus than bone	Bone and joint replacement, dental implants, dental restorations, heart valves
Stainless steels	316L stainless steel	High wear resistance [23]	Allergy consideration with Ni, Cr and Co [2] much higher modulus than bone	Fracture fixation, stents, surgical instruments
Others	Ni-Ti	Low Young’s modulus	Ni cause allergy [2]	Bone plates, stents, orthodontic wires
	Platinum and Pt-Ir	High corrosion resistant under extreme voltage potential and charge transfer conditions [30]		Electrodes
	Hg-Ag-Sn amalgam	Easy *in situ* formability to a desired shape susceptible to corrosion in the oral environment [30]	Concerns related to Hg toxicity [30]	Dental restorations

#### 3.1.4. Limitations of Current Metallic Biomaterials

The presence of elements such as Ni, Cr, and Co in both stainless steel and Co-Cr alloys has toxic effects [31]. Ni toxicity leads to dermatitis. The long-term existence of Al and V ions in Ti alloys has been found to cause Alzheimer’s disease, osteomalacia, and neuropathy in the long term [32]. The presence of Co has also been reported to have carcinogenic effects [33]. Recently, it is reported in [34] that stainless steels and Co-Cr alloys usually contain some harmful elements, such as Ni, Co, and Cr. In addition, 6Al-4V alloy is composed of cytotoxic elements like Al and V, which may cause severe problems once released inside the human body.

**Figure 1:**
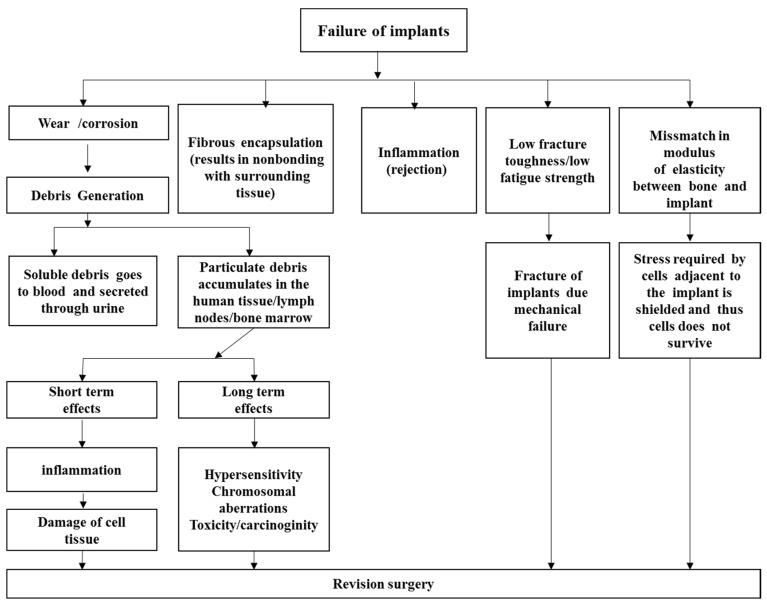
Reported causes of implant failure [18].

A high friction coefficient and wear debris formation can produce an inflammatory reaction, leading to the loosening of implants due to osteolysis [35]. A high modulus of elasticity leads to stress shielding, which causes implant failure. Figure 1 summarizes the reported causes of implant failure.

## 4. Wear of Metallic Biomaterials

The aforementioned discussion illustrates that the wear resistance of the biomaterial plays a significant role in the proper functioning of the material. Hence, the wear resistance of a biomaterial is clinically important. Several studies have been performed to investigate the tribological properties of developed biomaterials. We first summarize various wear test configurations that have been used by various researchers, followed by a description of the different techniques that are used to characterize wear and a review of various results for the wear and friction that have been obtained by different researchers.

### 4.1. Wear Testing Methods

**Figure 2:**
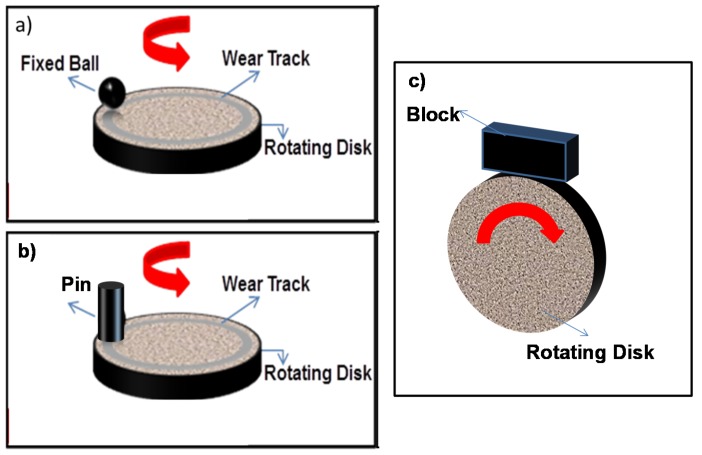
Schematic of (**a**) ball-on-disc, (**b**) pin-on-disc, and (**c**) block-on-disc wear test configurations.

Given the aforementioned limitations, especially in terms of the tribological properties, it is critical to characterize the wear and friction of developed biomaterials using a suitable test methodology. The methods that are most commonly used in the literature to study the tribological behavior of metallic biomaterials are the block-on-disc [36,37], ball-on-disc [38,39,40,41], and pin-on-disc [42], as shown in Figure 2. The temperature of the tests was selected to be 37 ± 0.1 °C to simulate real [37,38] or ambient [36] conditions. The wear tests were conducted in an environment of simulated body fluids (Ringer’s solution) [36] or fluids containing NaCl and phosphate-buffered solutions (PBS) [26]. However, in a few studies, tests were also conducted under dry sliding conditions [37,39]. Table 6 summarizes the advantages and disadvantages of various wear test configurations.

**Table 6:** Advantages and disadvantages of various wear test configurations [43].

Test	Advantages	Disadvantages
Pin-on-Disk	After run-in, surface pressure remains constant. Easy to determine wear volume and wear rate. The model closely simulates a linear friction bearing.	Difficult to align pin. If the pin does not stand perfectly vertical on the plate, edge contact results. A very long run-in time is therefore necessary. The front edge of the pin can skim off lubricant. This makes a defined lubrication state impossible.
Ball-on-Disk	High surface pressures are possible. The ball skims off lubricant less than a pin does. The model is similar to a linear friction bearing and a radial friction bearing.	Very small contact ratio: The contact surface of the ball is small compared to the sliding track on the disk. The contact area is enlarged by wear. Difficult to determine the wear volume of the ball.
Block-on-disc	The model is capable of simulating a variety of harsh field conditions, e.g., high temperature, high speed, and high loading pressure.	

### 4.2. Characterization Techniques for the Wear of Biomaterials

Different characterization techniques have been used to evaluate tribological behavior. Chemical analyses, such as energy-dispersive X-ray spectroscopy (EDXS/EDS), have been used for selected regions to determine the composition. A scanning electron microscope/ light microscope (SEM/LM) has been used to determine the wear mechanism or to perform a post-test examination [30]. A 3D Talysurf instrument has been used to measure the 3D surface roughness [38]. X-ray photoelectron spectroscopy (XPS) has been used to study the surface chemistry. The volume loss in the wear track has been measured using an optical profilometer/3D-profilometer. An atomic force microscope (AFM) has also recently been used [44] to study different tribological phenomena, such as friction, surface roughness, scratching, and adhesion. Moreover, interesting mechanical properties, such as the modulus of elasticity and hardness, could be measured using AFM with a depth-sensing indentation system.

### 4.3. Wear Performance of Different Biomaterials

Table 7 lists various developed materials and their fabrication techniques, the types of wear tests, and the parameters used to characterize wear, along with a summary of the results obtained by various researchers.

**Table 7:** Wear studies of metallic biomaterials.

First author, year	Material & fabrication processes	Experimental test techniques & parameters	Main results
Cvijovic’-Alagic *et al*. [36]	Ti-13Nb-13Zr Ti-6Al-4V Arc melting	A **block-on-disc** tribometer was used to conduct wear and friction tests in a simulated body fluid (Ringer’s solution). **Temperature**: ambient **Normal load**: 20–60 N **Sliding speed**: 0.26–1.0 m/s	The Ti-6Al-4V alloy showed a higher wear resistance than the Ti-13Nb-13Zr alloy. Abrasion was the primary wear mechanism.
Stefano Gialanella *et al*. [37]	NiTi Commercial alloy	A **block-on-disc** was used to measure dry sliding wear. A profilometer was used to quantify wear. **Sliding speed**: 0.837 ms^−1^ **Sliding distance**: 1004 m **Loads**: 50 to 200 N	A NiTi/WC-Co coupling exhibited a high wear rate. **Wear mechanism**: a transition from delamination wear to a regime featuring a mixture of delamination and oxidation wear.
K.S. Suresh *et al*. [38]	Ti-13Nb-13Zr Equal channel angular pressing (ECAE)	A tribometer was used as a lubricity fretting test system for texture and wear behavior; fretting wear and3D surface texture measurements were performed. **Normal loads**: 6 N **Frequency**: 20 Hz **Temperature**: 37 ± 0.1 °C	The grain size and the texture of material affected the wear of the surface. There was no difference in the friction coefficient between the ECAE processed and as-received samples.
Li-juan Xu *et al*. [39]	β-type Ti-15Mo-xNb arc-melting vacuum-pressure casting system	A **ball-on-disc** was used for dry wear tests. **Normal load**: 1 N and 2 N **Test-disc rate**: 100 r/min	The lowest friction coefficient was obtained for a Ti-15Mo-5Nb alloy under a 1-N load. Adhesion was the primary wear mechanism.
M. Fellah *et al*. [41]	Ti-6Al-7Nb and AISI 316L stainless steel	**Ball-on–disc** and sphere-on-plane **Load**: 3 N, 6 N and 10 N **Sliding speed**: 1 mm/s, 15 mm/s and 25 mm/s	The same mechanisms of wear and friction were found for all of the tested samples.
S.J. Li *et al*. [42]	Ti-Nb-Ta-Zr and Ti-6Al-4V induction skull melting method	**Reciprocal pin-on-disc** in a 0.9% NaCl solution **Reciprocating velocity**: 45 rpm **Sliding distance**: 30 km	The wear resistance of Ti-29Nb-13Ta-4.6Zr was enhanced by incorporating Nb_2_O_5_ oxide particles into the diffusion-hardened surface of the alloy.
Animesh Choubey *et al*. [45]	CP Titanium, Ti-6Al-4V, Ti-5Al-2.5Fe, Ti-13Nb-13Zr and Co-28Cr-6Mo	**Ball on flat fretting** wear tester: Hanks’ balanced salt solution **Normal load**: 10 N for 10,000 cycles **Frequency**: 10 Hz	The primary wear mechanisms of Ti alloys were tribomechanical abrasion, transfer layer formation and cracking.
A. Iwabuchi *et al*. [46]	Co-29Cr-6Mo alloy and Ti-6Al-4V	**Fretting apparatus and a reciprocating** sliding tribometer: Quasi-body fluid, Hanks’s solution. **Normal load**: 5 N; **frequency**: 10 Hz **Temperature** in the solutions: 37 ± 2 °C	Co alloy exhibited good wear resistance; Ti alloy exhibited good fretting resistance.
X. Luo *et al*. [47]	ASTM F1537 Co-Cr alloy	**Pin-on-disc** tribometer **Load**: 20 N **Rotation speed**: 60 (rpm)	The tribocorrosion properties of the Co-Cr alloy were enhanced by a layer of the S-phase.
Akihiko Chiba *et al*. [48]	Co-Cr-Mo forged	**Pin-on-disc** **Load**: 9.8 N 24 rpm	Forged CoCr exhibited a lower wear loss than a cast CoCr alloy.
S. M. T. Chan *et al.* [49]	(CoCr), stainless steel (SS)	**Pin-on-disc** **sliding speed**: 0.5 mm/s, 5 mm wear track radius **Normal load**: 1.8 N	
Alfons Fischer *et al.* [50]	AISI 316L CoCr29Mo6	**Pin-on-disc** for dry sliding wear tests **Load**: 5 N **Relative velocity**: 0.1 m/s **Ambient temperature**: 25 °C	Ni-free high-nitrogen steel and LC-CoCrMo alloy exhibited higher wear resistance and dry friction than Ni-containing austenitic steels.
A. Igual Muñoz *et al.* [40]	Co-Cr-Mo Low and high carbon	**Tribocorrosion techniques** **Load**: 1.2 N; **frequency**: 1 Hz **Temperature**: 37 ± 0.1 °C Simulated body fluids [NaCl and phosphate-buffered solutions (PBS) with and without albumin]	LC CoCrMo had a higher wear resistance in NaCl and PBS albumin than HC. No differences were observed for the alloys in the other solutions.
M. Alvarez-Vera, *et al.* [51]	Co-Cr alloy with boron additions (0, 0.3, 0.6 and 1 B wt%) by casting method	three-axial hip joint simulator	Wear resistance as the boron increased.
L. Mohan, *et al.* [52]	Commercial Ti-13Nb-3Zr alloy oxygen implanted	Reciprocating type wear tester normal forces: 3, 5 and 10 N. The stroke length: 10 mm and an alumina ball of 6 mm diameter was used as the counter surface	The implanted samples display a lower friction coefficient as compared to the substrate one.
H. Attar *et al*. [53]	Commercially pure titanium (CP-Ti) parts produced using selective laser melting (SLM) and casting	a pin-on-disc at room temperature A stainless steel disc of 45 mm diameter loads: (15 N, 20 N,25 N and 30 N) sliding speed: 0.5 m/s for 15 min.	SLM CP-Ti showed better wear resistance compared to casting as a result of fine grains and higher microhardness.

**Figure 3:**
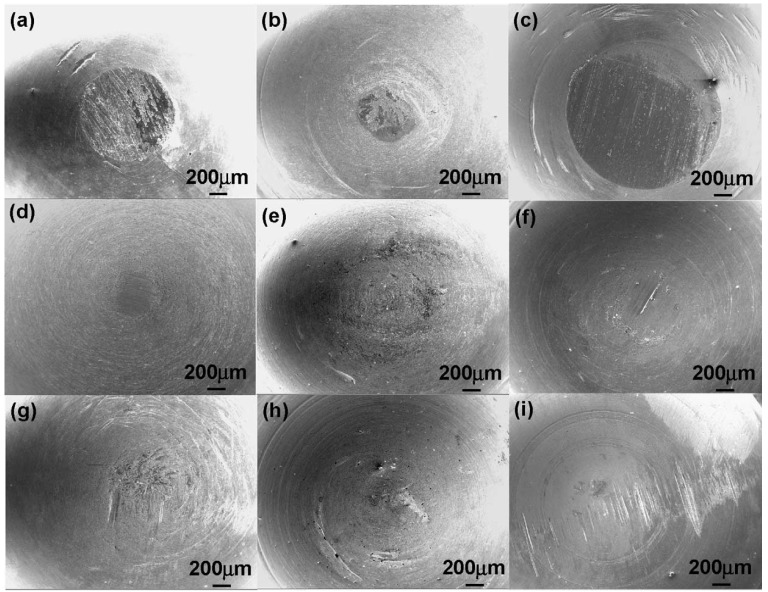
Wear morphology of (**a**) TNZT1, (**b**) TNZT3, and **(c**) TAV1 sliding on a stainless steel plate; (**d**) TNZT1, (**e**) TNZT3, and (**f**) TAV1 sliding on UHMWPE; (**g**) TNZT1, (**h**) TNZT3, and (**i**) TAV1 sliding on a pig bone in 0.9% NaCl [36].

Li *et al*. [42] studied the effects of the Nb content, surface modification, the material of the counterface, and heat treatment on the wear characteristics of Ti-Nb-Ta-Zr and Ti-6Al-4V (TAV1) alloys. The authors found that increasing the Nb content improved the wear resistance. Heat treatment enhanced the resistance to wear of Ti-29Nb-13Ta-4.6Zr (TNZT1) because of the formation of oxide particles from Nb_2_O_5_. The material of the counterface was reported to have a significant effect on the wear loss. Although no wear was observed for the sliding of polyethylene (UHMWPE) and a pig bone on the oxidized surface of Ti-29Nb-13Ta-4.6Zr, the wear loss from the sliding of these materials on stainless steel was higher than on both the TNZT1 and TAV1 alloys. Figure 3 compares the morphology for different alloys sliding against different counterfaces.

**Figure 4:**
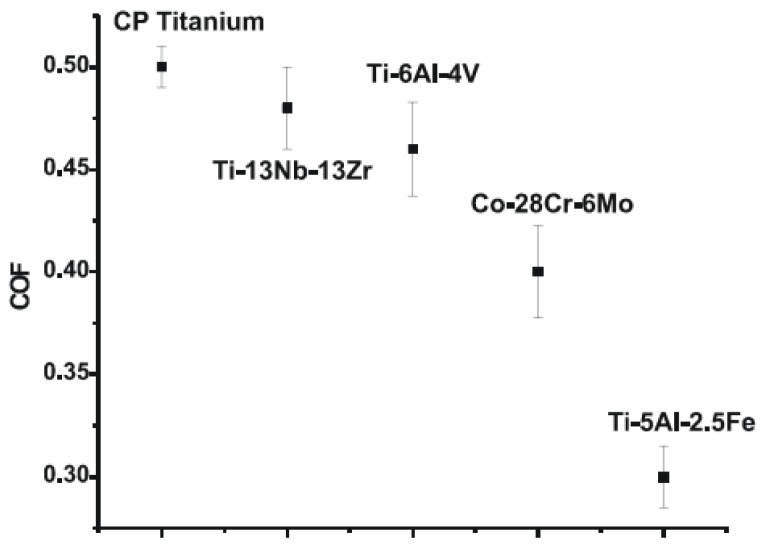
Steady-state friction coefficient for the studied materials [45].

Choubey *et al*. [45] investigated the tribological characteristics of commercially pure (CP) Ti, Ti-6Al-4V, and Ti-5Al-2.5Fe in Hanks’s solution (a simulated body fluid solution). The observed wear mechanisms were tribo-mechanical abrasion, transfer layer formation, and cracking. The predominant wear mechanism was tribo-mechanical wear. Figure 4 compares the steady-state coefficient of friction (COF) for the studied materials. The COF of Co-28Cr-6Mo under fretting was 0.4, whereas a superior (lower) value of 0.3 was observed for a Ti-5Al-2.5 Fe/steel couple.

Cvijovic’-Alagic *et al*. [36] compared the tribological behavior of Ti-13Nb-13Zr and Ti-6Al-4V ELI under different heat treatment conditions. The results showed that the wear resistance of the Ti-6Al-4V ELI alloy was superior to that of the Ti-13Nb-13Zr alloy under all of the heat treatment conditions. The martensitic microstructure of the WQ Ti-6Al-4V ELI alloy increased the hardness of the alloy, resulting in superior resistance to plastic deformation during the wear test. The wear mechanism observed for this alloy was predominantly abrasion. The lower hardness of the Ti-13Nb-13Zr alloy resulted in a higher amount of wear loss than for the Ti-6Al-4V alloy.

Suresh *et al*. [38] studied the wear behavior of an ultra-fine grained (UFG) Ti-13Nb-Zr alloy. The samples were processed by equal channel angular extrusion (ECAE). The effect of the surface roughness on the wear behavior was also studied. The authors concluded that both the grain size and surface texture affected the mechanical properties and wear of the as-received alloy. However, there was no significant difference between the average friction coefficient of the ECAE-processed alloy and that of the as-received sample. In addition, no major change was observed in the fretting wear after ECAE processing for both samples. This result was attributed to the absence of a significant increase in the hardness of the samples after ECAE. Abrasion was observed to be the predominant wear mechanism. A few researchers [54,55] reported that the UFG nanostructured materials increase the hardness and lead to enhancement in the wear resistance and resistance to wear debris formation. The UFG materials obtained by the severe plastic deformation (SPD) process exhibit lower friction coefficients and better wear resistance [56]. UFG Ti samples in two processing states—equal channel angular pressing (ECAP) for eight passes and ECAP + further cold rolling for 75% strain—were studied and the results showed that the UFG structure results in a lower adhesion component and consequently lowers friction coefficient, which in turn improved the wear property [56].

Xu *et al*. [39] investigated the wear resistance of Ti-15Mo-xNb (x = 5%, 10%, 15%, and 20%) alloys under dry conditions. The friction coefficient was observed to increase with the Nb content. Adhesive wear was observed to be the primary wear mechanism.

**Figure 5:**
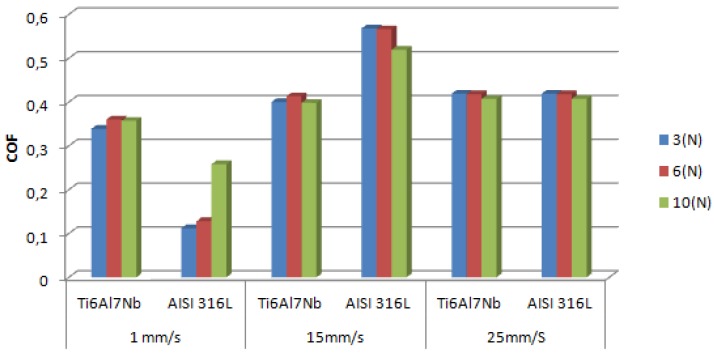
Mean friction coefficients of AISI 316L and Ti-6Al-7Nb [41].

Fellah *et al*. [41] examined the wear behavior of Ti-6Al-7Nb and AISI 316L stainless steel alloys at different sliding speeds and loads. Figure 5 compares the mean values of the friction coefficient for both alloy systems under different conditions. The same friction and wear mechanisms were observed for the tested samples. The change in the wear rate with sliding speed was not significant for the Ti-6Al-7Nb alloy. An increase in the friction coefficient with the sliding speed was observed for both alloys. The wear mechanisms that were observed at high speed were plastic deformation and adhesive wear.

**Figure 6:**
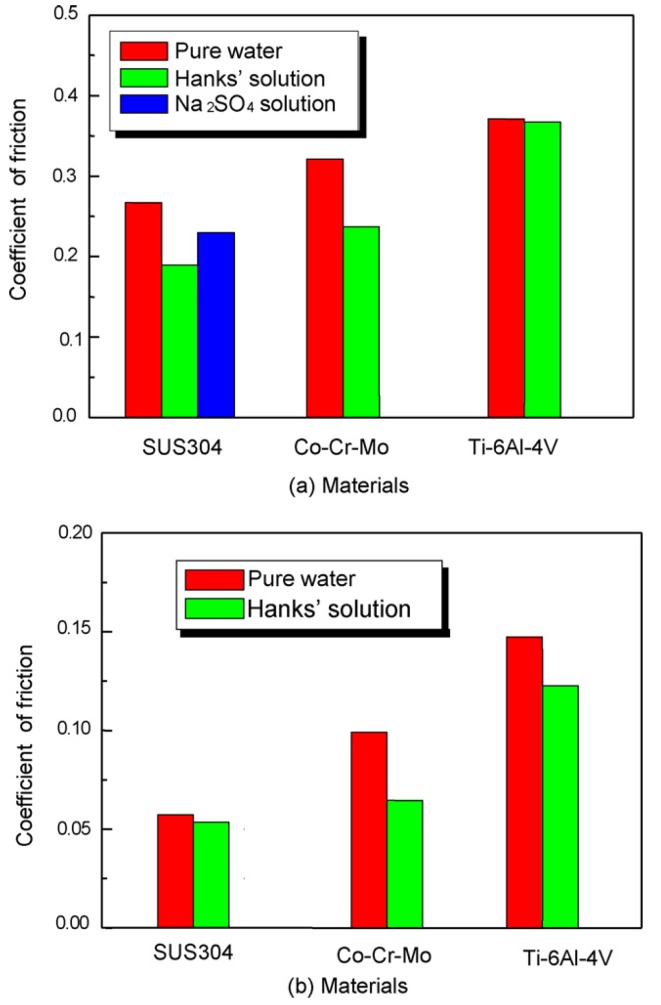
Mean friction coefficient against an Al_2_O_3_ ball in (**a**) fretting and (**b**) sliding [46].

Iwabuchi *et al*. [46] conducted a fretting wear test and a reciprocating sliding wear test on a Co-29Cr-6Mo alloy, a Ti-6Al-4V alloy, and a SUS304 stainless steel alloy in Hanks’s solution. The results showed the good fretting resistance of the Ti-6Al-4V alloy relative to that of the standard SUS304 stainless steel alloy, whereas the corresponding resistance to sliding wear was poor because of abrasion. The Co-29Cr-6Mo alloy exhibited good wear resistance, and the synergy effect was considerably stronger in the fretting test than in the sliding wear test. Figure 6 compares the mean value of the friction coefficient in pure water to that in Hanks’s solution. The figure clearly shows higher mean values for the friction coefficient in the fretting test than the sliding test for both liquids.

**Figure 7:**
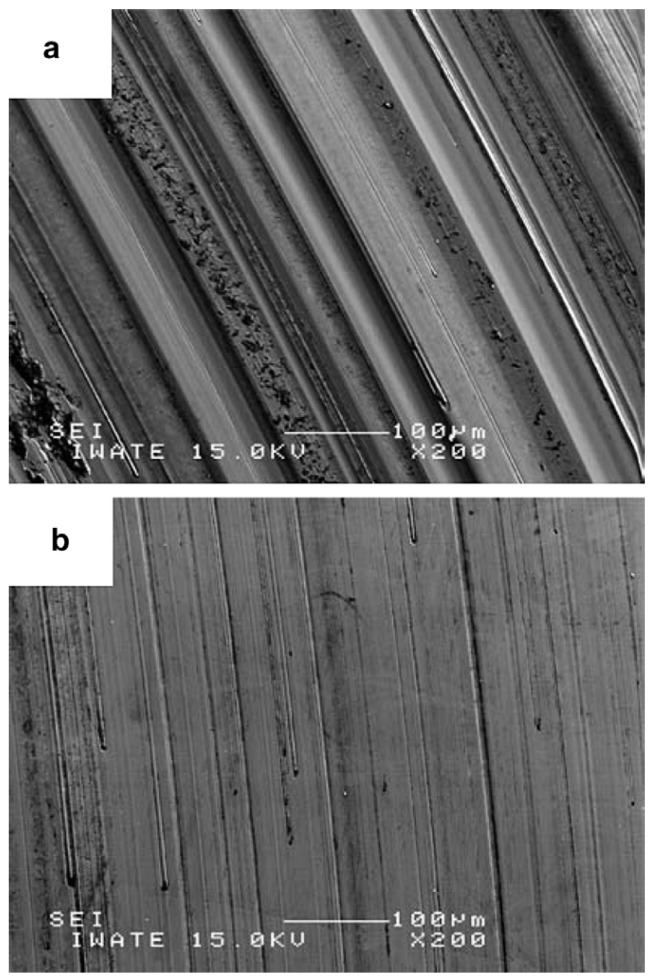
SEM micrographs of wear scars of (**a**) cast CoCr alloy and (**b**) forged CoCr alloy [48].

Chiba *et al*. [48] studied the wear characteristics of a forged Co-Cr-Mo alloy and a cast CoCr alloy with high carbon contents. The results showed that the wear resistance of the forged CoCr alloy was higher than that of the cast CoCr alloy. The higher wear loss of the cast CoCr alloy resulted from the precipitation of carbide in this alloy. Figure 7 is a comparison of the SEM micrographs of the wear scars on both surfaces. The number of grooves and scratches in the cast CoCr alloy was higher than that in the forged CoCr alloy. The measured friction coefficient of the forged alloy was higher than that of the cast alloy.

**Figure 8:**
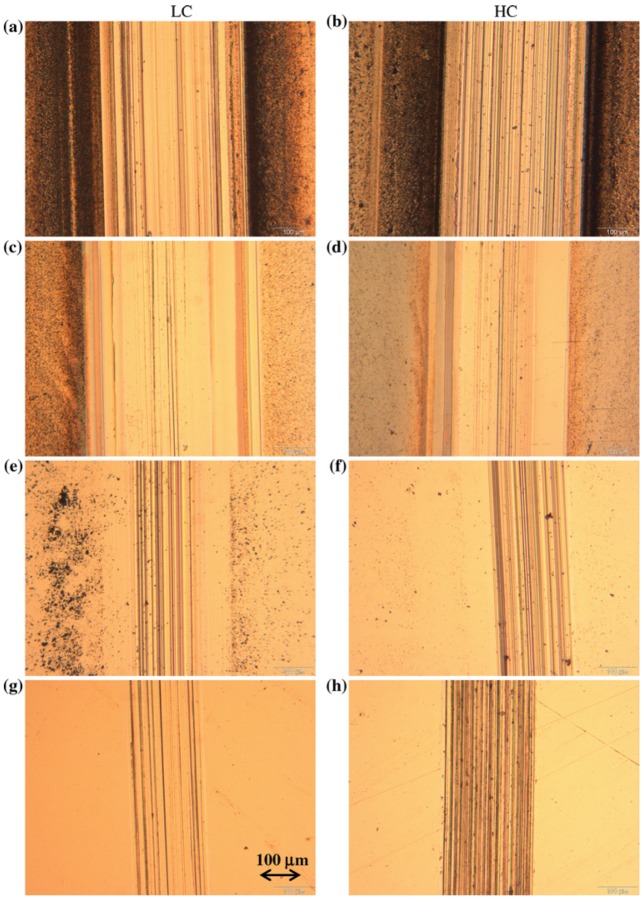
Optical microscopy images of wear trace LC and HC alloys during sliding in (**a**) and (**b**) NaCl; (**c**) and (**d**) NaCl with albumin; (**e**) and (**f**) PBS; and (**g**) and (**h**) PBS with albumin [40].

Muñoz *et al*. [40] used tribo-corrosion to compare the wear of CoCrMo alloys in two forms—low carbon (LC) and high carbon (HC)—in four different simulated body fluids. The results showed that the wear behavior of these alloys depended on the surrounding environment. There was no difference in the wear resistance between the LC and HC alloys in both NaCl and PBS without albumin. The LC alloy had a higher wear resistance than the HC alloy in NaCl and PBS with albumin. The difference in the alloy behavior under different solutions was attributed to the enhancement of corrosion from sliding and chemical effects at the surface that affected both the mechanical wear and third-body behavior. Figure 8 compares the wear tracks for both the LC and HC alloys under different solution conditions. The optical microscope images clearly show scratches over the scar length, illustrating that abrasion was the predominant wear mechanism. The wear debris accumulated around the track for both alloys in the NaCl solution and was higher than in the PBS solution. Interestingly, the presence of albumin in the solutions reduced the wear debris for both alloys.

Fischer *et al*. [50] examined the wear characteristics of CrNiMo steels, low-carbon Co-base alloy CoCr29Mo, CrNiMnMoN steel, and Ni-free CrMnMoN steel alloys in sliding wear. They evaluated the wear characteristics in sliding wear using a pin-on-disk tribometer and torsional fatigue tests followed by electron microscopy. The results showed that Ni-free CrMnMo exhibited superior wear behavior compared to CrNiMo steels. This improvement was found to result from planar slip because of the formation of nano-sized wear particles in the CrMnMo alloy containing C and N. However, the CrNiMo containing Ni exhibited wavy-slip. The wear particles that formed were either nano- or micron-sized and had a higher wear rate than that of Ni-free high-nitrogen steel and the LC CoCrMo alloy. This difference in the wear rate was attributed to the tribo-chemical reactions of the nano-sized wear particles, which resulted in surface nano-fatigue. In contrast, the micron-sized wear particles produced abrasion, micro-fatigue, and micro-ploughing that inhibited tribo-chemical reactions.

By decreasing the length-scale of the contact, the hardness and yield stress will increase [57,58,59]. Therefore, it is important to study the tribological and mechanical properties of the biomaterials at the relevant scale.

Beake *et al*. [60], studied the nano-scratch and nano-fretting of Ti6Al4V, 316L stainless steel and CoCr alloy. The nano-scratch and nano-fretting tests were performed with a commercial nanomechanical test system. Tests were performed at 25 °C using a 3.7 mm sphero-conical diamond indenter. The results showed that the CoCr alloy possesses better wear resistance over a wide range of experimental conditions compared to Ti6Al4V, and 316L stainless steel alloys exhibited decreasing wear resistance with an increase in the fretting load.

Sun *et al*. [61], investigated the Micro-abrasion mechanisms of cast CoCrMo in simulated body fluids. A modified Phoenix Tribology TE/66 micro-abrasion tester was semi immersed in a liquid tank. Moreover, the nanoindentation and nano-scratch test were performed to study the nano-scale material deformation occurring during micro abrasion process. The results showed that the abrasive wear rate and wear mechanisms of the CoCrMo are dependent on the nature of the third body abrasives, their entrainment into the contact, and the presence of the proteins. The interaction between the specimen and the abrasive was affected by the presence of protein due to its influence on the solution viscosity. The existence of protein at lower abrasive volume fractions acts as a boundary lubricant and reduces the wear loss; however, at a high volume fraction of abrasive, the existence of protein enhanced the wear loss.

This study is important for *in vivo* wear corrosion study of the alloys in the hip joint as the hard particles sizes are small and less in quantity.

### 4.4. Techniques to Improve Wear Resistance of Metallic Biomaterials

In addition to the various processing techniques and compositional changes that enhance the wear resistance of metallic biomaterials, the following surface modification techniques for improving the wear resistance of the biomaterials have also been reported in the literature.

- **Ion implantation (physical deposition)** is considered a simple technique for significantly modifying the physical and/or chemical properties of the near surface of a material in which suitable ions are embedded into the surface of a material from a beam of ionized particles. This technique has been reported to improve the wear performance of Ti6Al4V and Co28Cr6Mo alloys [62].
- **Nitriding** (a thermo-chemical surface treatment) has been used to increase the resistance of a Ti6Al4V alloy to dry wear [63,64].
- **Carburization and boriding** techniques are used to enhance surface hardness, which in turn improves the wear resistance.
- **Plasma spray coating** has also been used to enhance the wear resistance of few biomaterials.

## 5. Summary

An extensive literature survey has shown that the wear resistance of Ti and Co alloys has been improved under different conditions in extensive studies. We summarize the results of these studies for Ti- and Co-based metallic biomaterials below.

**Ti alloys**
  - In general, adding Nb to Ti alloys enhances the wear resistance of these alloys and slightly increases the friction coefficient primarily because of the increase in the hardness of the alloy. The heat treatment of these alloys has been observed to further increase the wear resistance because of the formation of Nb_2_O_5_ particles.
  - Abrasive wear has been observed to be the predominant wear mechanism.
  - Hence, surface treatments and coating are necessary to enhance the resistance of alloys to wear and friction.
**Co alloys**
  - Forged CoCr alloys exhibit higher wear resistance than cast CoCr alloys. However, the friction coefficient of the forged CoCr alloys has been observed to be higher than that of the cast alloys.
  - The wear behavior of LC and HC CoCrMo alloys depends on the surrounding environment.
  - Ni-free CrMnMo exhibits improved wear behavior compared to CrNiMo steels.

It is important to study the tribological and mechanical properties of the biomaterials at the relevant scale.
